# Supplementary material for: Rational engineering of allosteric protein switches by in silico prediction of domain insertion sites
Source: Nat Methods. 2025 Aug 4;22(8):1698–706. doi: 10.1038/s41592-025-02741-z (PMC12328240; doi:10.1038/s41592-025-02741-z)
Supplement: Supplementary file 1 — Supplementary Notes 1 and 2, Figs. 1–17 and Tables 1–4. [file 41592_2025_2741_MOESM1_ESM.pdf]

nature methods

Supplementary information

https://doi.org/10.1038/s41592-025-02741-z

Rational engineering of allosteric protein switches by in silico prediction of domain insertion sites

In the format provided by the authors and unedited

Contents:

Supplementary Note 1-2
Supplementary Figures 1-17
Supplementary Tables 1-4

Supplementary Note 1 | Details on model training and optimization.

Having built a large domain insertion dataset, we aimed to employ it for machine learning to predict sites in proteins suitable for domain insertion and thus for protein engineering. To this end, we removed the insert domain sequence portion from each protein in our data set. This resulted in semi-synthetic sequences of proteins lacking their naturally occurring inserts (parent_only). Importantly, for all parent_only proteins, we have information about the true domain insertion site. Analysis of the secondary structure around the insertion sites revealed enrichment for coils (~50%) as compared to α-helices (~35%) and β-sheets (~15%) (Supplementary Fig. 3). This site and its two adjacent positions were considered as positive labels for model training, since exact domain boundaries are difficult to define and previous experimental work has shown that domain insertions are often tolerated at several consecutive sequence positions[1,2]. All other sequence positions were assumed not to tolerate domain fusion and were therefore assigned negative labels. Importantly, this assumption is rather naive, as some of these positions may, in fact, tolerate domain fusion (see below). That said, due to the lack of further information on these sites and in light of experimental evidence that most positions in proteins do not tolerate domain fusion[1,2], this label assignment is a reasonable basis for model training (Fig. 2a).

Subsequently, the dataset was randomly split into train validation and test sets (ratio 70:20:10; referred to as “random” split). Since simple one-hot encoding of sequences was, as expected, not suitable for model training (Fig. 2b and Supplementary Fig. 4a), we computed information-rich multidimensional representations (embeddings) of the parent_only protein sequences with ESM-2[3]. Based on these embeddings, we then trained multi-layer perceptron (MLP) decoders to identify the true domain insertion sites in the parent_only sequences. The resulting model accurately inferred true insertion sites with a median probability score of 0.9, while reliably assigning low scores to negatively labeled positions (median score = 4.32e-06; Fig. 2b and Supplementary Fig. 4b). We further investigated whether scaling the decoder to more complex architectures would yield a significant performance improvement. Indeed, replacing the MLP with BERT architectures of either 36.7 or 146.0 million parameters improved discrimination between positive and negative labels, mainly due to increased prediction scores for the positive positions[4] (Supplementary Fig. 4b).

The near-perfect performance on the validation set raised the question of whether our model had simply memorized information about suitable insertion sites between different members of the same domain superfamily present in the training and test sets, regardless of the strict sequence similarity cutoff we had used. To enforce generalization beyond the training data, we first split the data set by Interpro/CATH superfamily terms (referred to as “Interpro” split), meaning that members of the same superfamily occur in only one of the three subsets. To create an even more stringent dataset split, we randomly selected only one example sequence

for each parent domain superfamily, resulting in a much smaller dataset of only 232 highly diverse sequences (referred to as “single” split). We trained our MLP decoder again on these new datasets and compared all three training regimes on an intersected test set (Fig. 2c, Supplementary Fig. 4b). While we observed a greater overlap between the predicted probability distributions, both new models were generally still able to discriminate between insertion tolerant and non-tolerant sites (Fig. 2c).

To further optimize our approach, we considered two unique features of our dataset. First, the data is by definition highly unbalanced, with only three consecutive positive sites per parent_only sequence, which biases any model toward global prediction of low values (i.e. a trivial model that predicts only zeros has a high overall success rate). Second, we only have reliable information about the positive labels, whereas we do not know whether domain insertions would in fact be tolerated at some sites that are assumed to be negative.

To account for these factors, we introduced a masking strategy during loss computation that considers only the positive labels and a single, randomly selected, negatively labeled site of each parent_only sequence in each training epoch. This approach significantly increases the weight of the positive labels. Importantly, as the negative site is randomly selected in each epoch, the model still captures information about the entire protein sequence as training progresses. We assumed that this approach would facilitate the model to transfer the patterns learned on the true positive sites to the unknown protein regions, since the model would be less penalized in this regard during training. When we tested this strategy on all three dataset splits (random, Interpro, single), we observed successful discrimination between positive and negative labels in all cases (Fig. 2d). Importantly, models based on both the Interpro and single superfamily data splits, robustly predicted high scores for the true positives (median prediction of 0.46 (Interpro) and 0.87 (single) compared to 0.002 and 0.3 for negative labels, respectively; compare Fig. 2c and 2d). As intended, the model predicted approximately 10% of sequence positions as positives in regions of the parent_only protein, now including sites for which we had no information on domain insertion tolerance. The frequency of positives is also in line with our expectation derived from previous experimental observations[1,2]. We selected our model trained on the most restrictive, single sequence data split for further investigation and termed it ProDomino.

Supplementary Note 2 | Comparison of ProDomino to previous gradient boosting classifier approaches

We further compared ProDomino to a machine learning approach based on gradient boosting classifier models, which we had reported in 2023 (we refer to it as "Mathony et al." model)[1]. In this prior work, we had trained models on subsets of insertion mutagenesis data, i.e. these models inherently depend on experimental data.

Firstly, we asked how many randomly selected experimental data points would need to be provided to our gradient boosting classifier during training for it to reach the predictive performance of ProDomino, which operates independently of experimental data (ProDomino baseline). Using the available AraC[1] and *S. pyogenes* Cas9[5] insertion mutagenesis datasets as example (see main text, Fig. 4, and Supplementary Fig. 13), we determined that the gradient boosting classifiers need to be trained, on average, on >20 (for AraC) and >300 (for Cas9) data points to match ProDomino's performance (Supplementary Fig. 7).

Next, we asked how the performance of ProDomino would improve if we revealed experimental data for a specific target protein, again using AraC and Cas9 as examples. We therefore fine-tuned ProDomino on randomly selected sets of experimental data points and evaluated model performance on the remaining data not employed for fine-tuning - akin the training of the gradient boosting classifiers. The resulting ProDomino variants consistently outperformed the Mathony et al. models, irrespective of the number of data points revealed to both models in the process (Supplementary Fig. 7).

These findings underscore the robustness of ProDomino's predictive capabilities and its advantage, particularly in scenarios where experimental data is scarce or unavailable (which is the typical case). Moreover, the fact that we can boost ProDomino's performance by fine-tuning it on some experimental data points suggests that its underlying architecture enables more efficient generalization and feature learning, rendering it well-suitable for low-N engineering approaches.

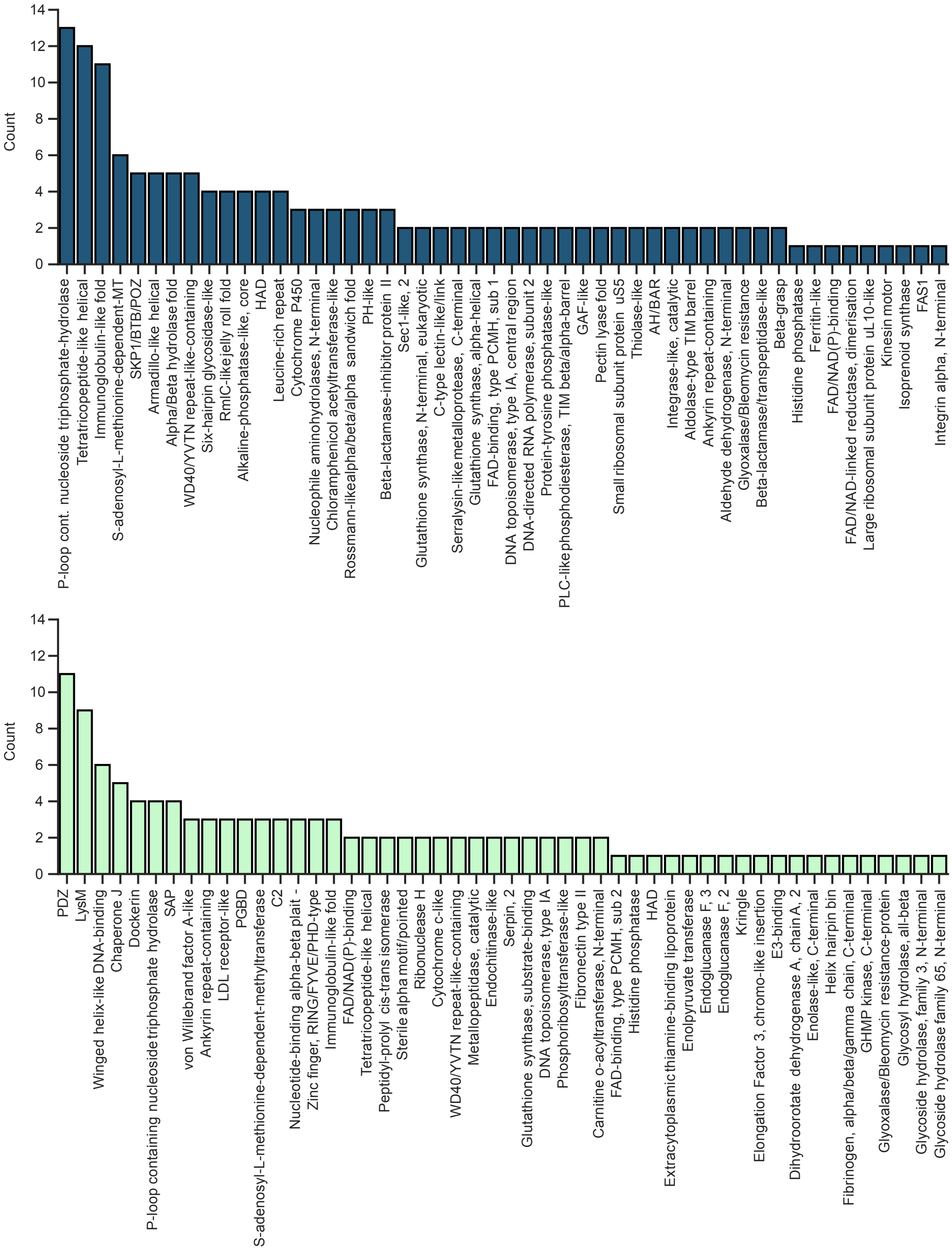

Supplementary Fig. 1 | Parent and insert domains show different degrees of promiscuity with respect to their fusion partners. The number of unique domain superfamily combinations is shown for a given parent (top panel) or insert domain (bottom panel) for top 50 domain superfamilies. The data correspond to Fig. 1c.

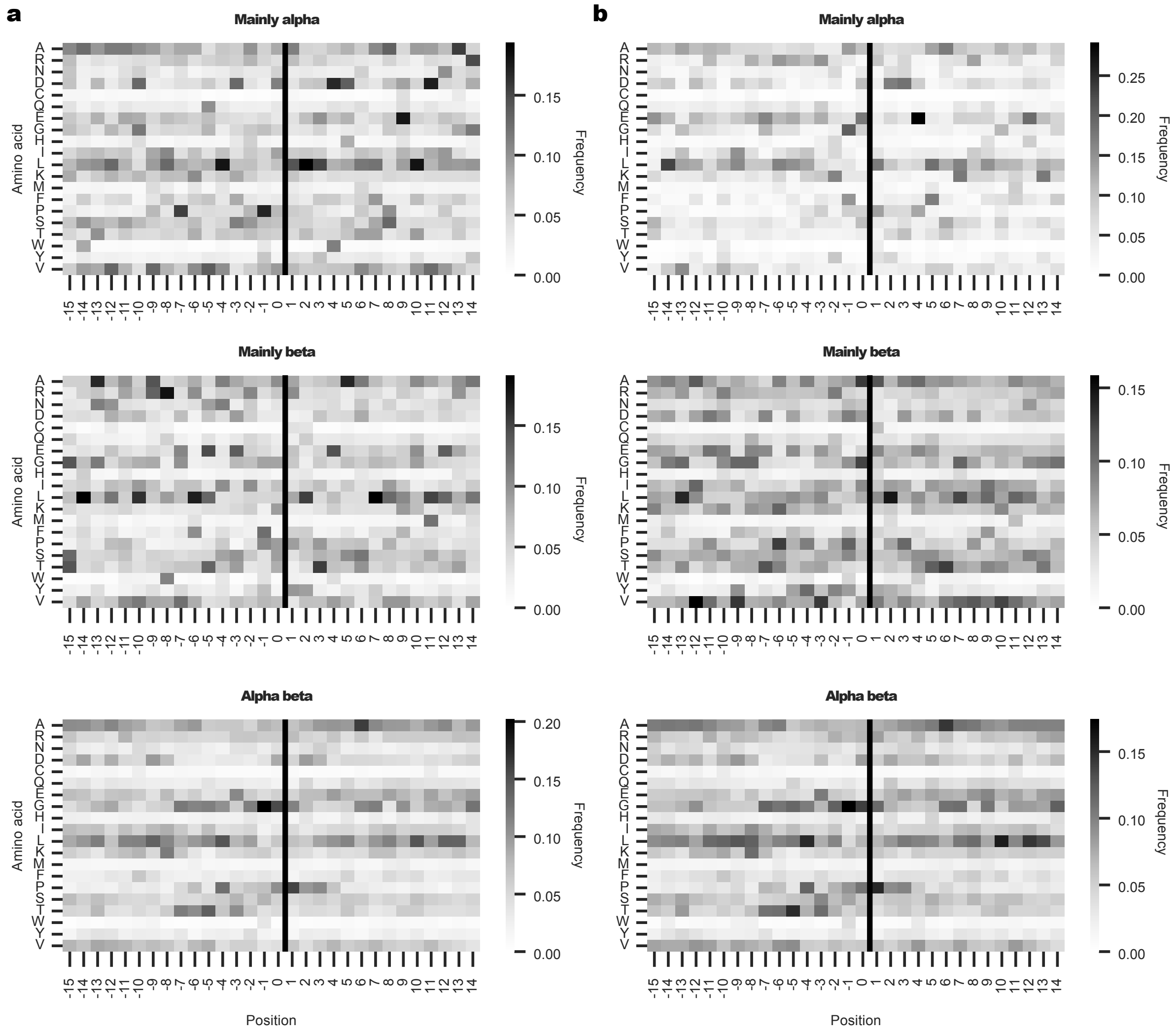

Supplementary Fig. 2 | Natural domain insertion sites do not show enrichment for specific sequence motifs. a, **b** Heatmaps of amino acid frequencies surrounding domain insertion sites are shown for different protein groups within the dataset. Heatmaps were generated for all parent domains belonging to the indicated CATH class (mainly alpha, mainly beta or alpha-beta) (**a**) and for all inserts within these classes (**b**). Black lines indicate domain insertion sites.

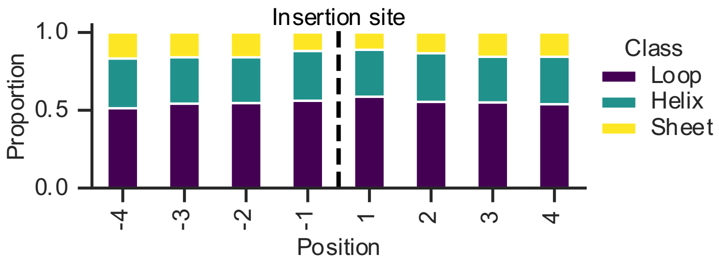

Supplementary Fig. 3 | Secondary structures around natural insertion sites. S4pred was applied to predict secondary structure elements of the semi-synthetic proteins around the true insertion site for the “single” dataset (refer to Supplementary Note 1).

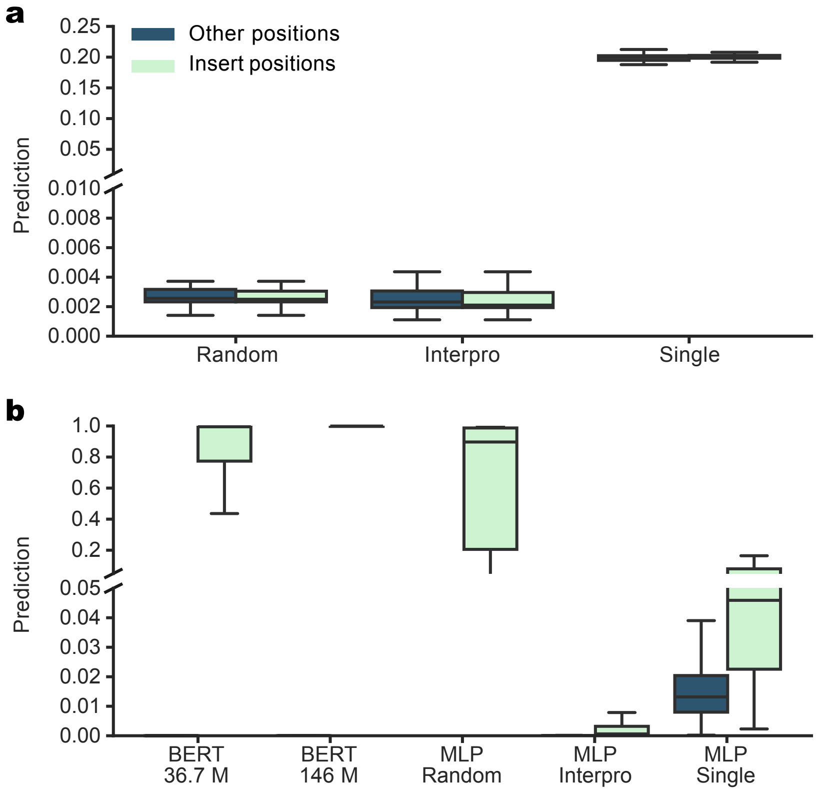

Supplementary Fig. 4 | Optimization of ProDomino models. a, Box plots show the test set performance of additional baseline MLP models trained on one-hot encodings (corresponds to Fig. 2c). **b**, Performance of BERT-like classifiers on the test set is shown. Box plots of the corresponding MLP classifiers from Fig. 2c are shown for comparison. **a**,**b** Boxes represent the interquartile range (IQR) and the median is represented by a horizontal line. Whiskers extend to the 1.5-fold IQR or to the value of the smallest or largest prediction value.

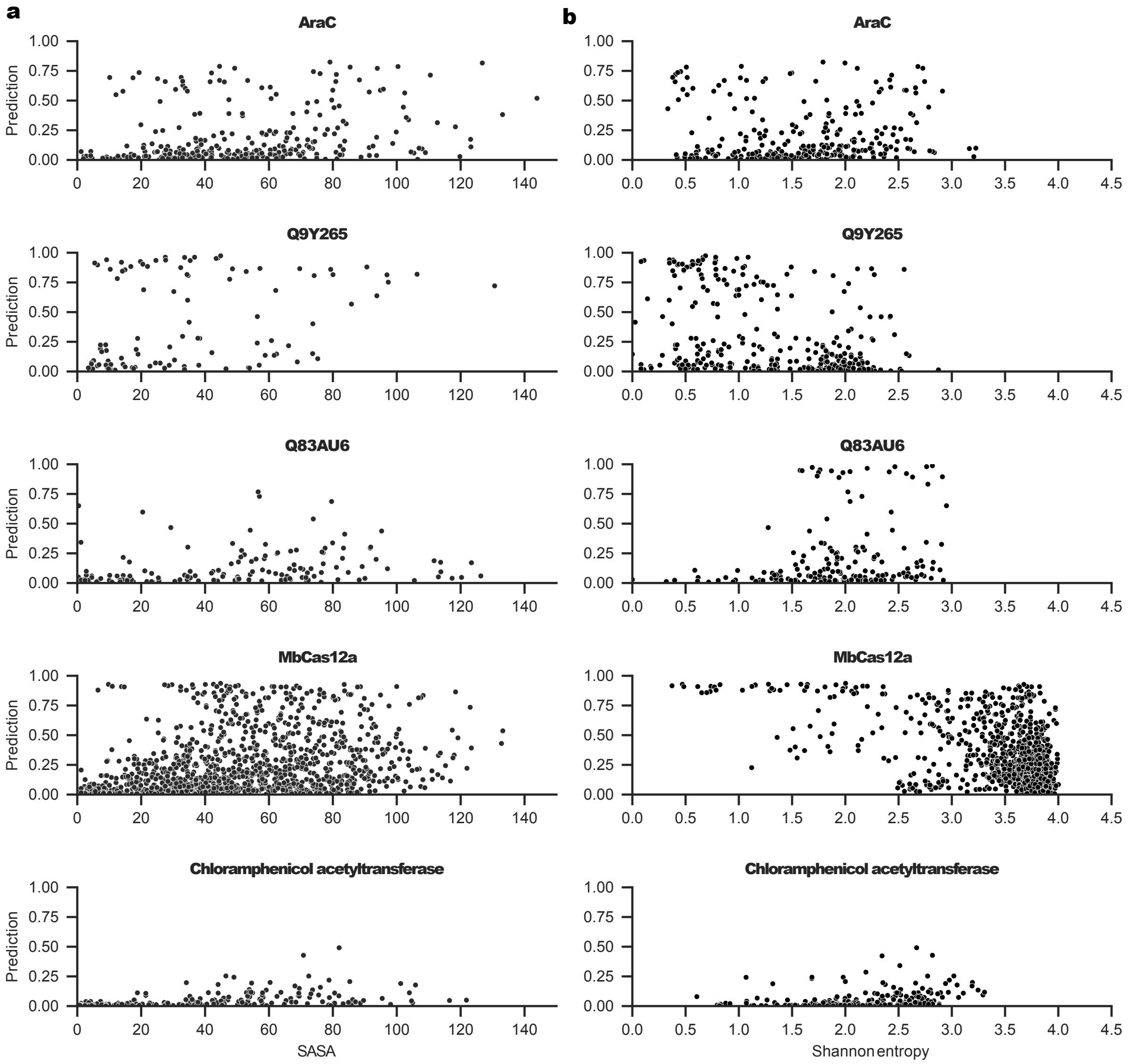

Supplementary Fig. 5 | ProDomino predictions do not correlate with SASA or sequence conservation. a, Scatterplots of the relation between ProDomino predictions and SASA for sample proteins with available structures. **b**, Scatterplots of the relation between ProDomino predictions and sequence conservation, represented by Shannon entropy, for selected proteins.

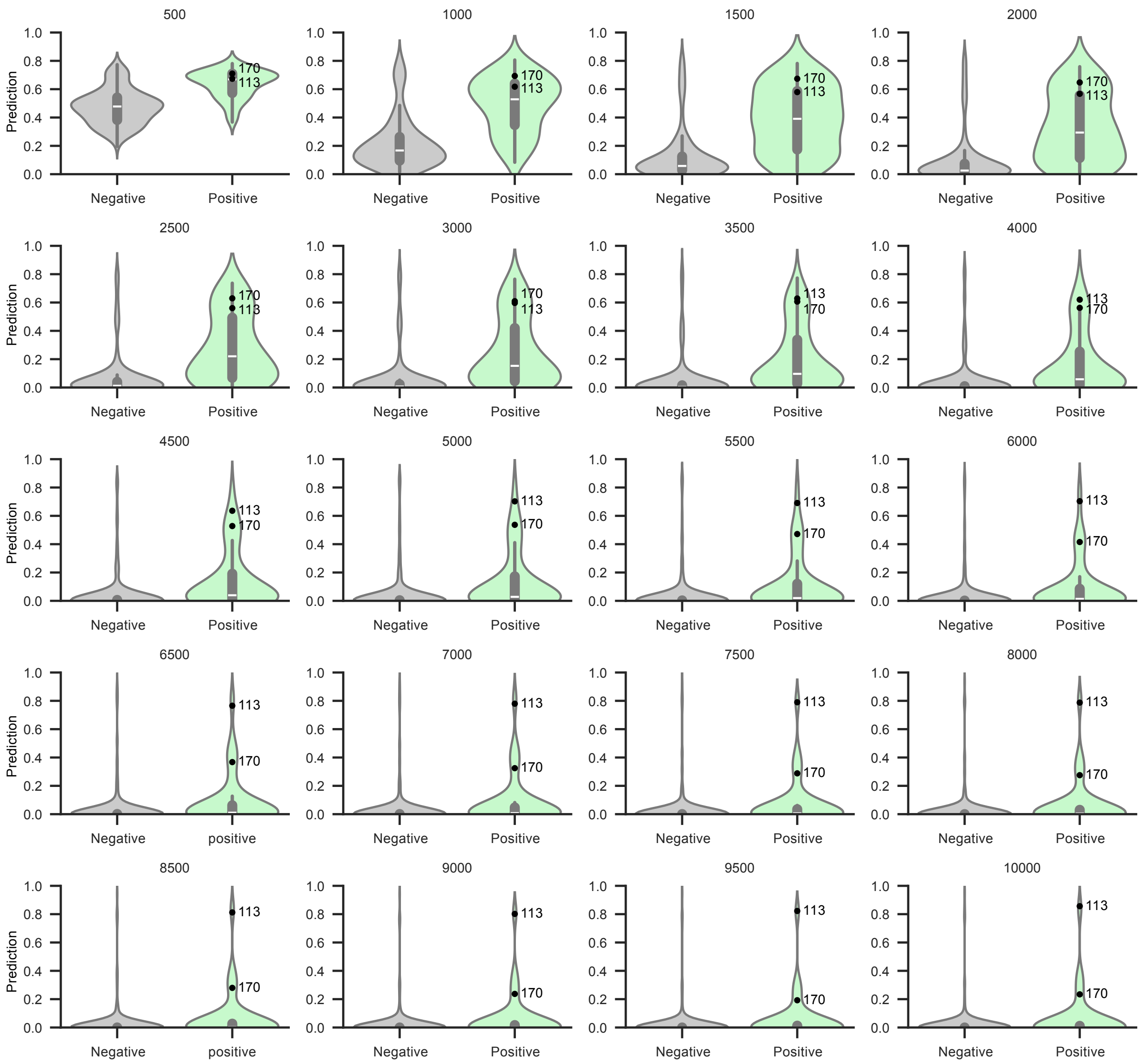

Supplementary Fig. 6 | ProDomino identifies allosteric sites previously used to engineer switchable AraC variants. Violin plots showing the distribution of predictions for insertion tolerant sites (green) and positions not amenable to domain insertion (gray). The scores for the insertion sites I113 and S170, which are known to give rise to light-regulated AraC variants when a LOV2 photosensory domain is introduced, are indicated. Boxes represent the interquartile range (IQR) and the median is represented by a white horizontal line. Whiskers extend to the 1.5-fold IQR or to the value of the lowest or highest predicted value.

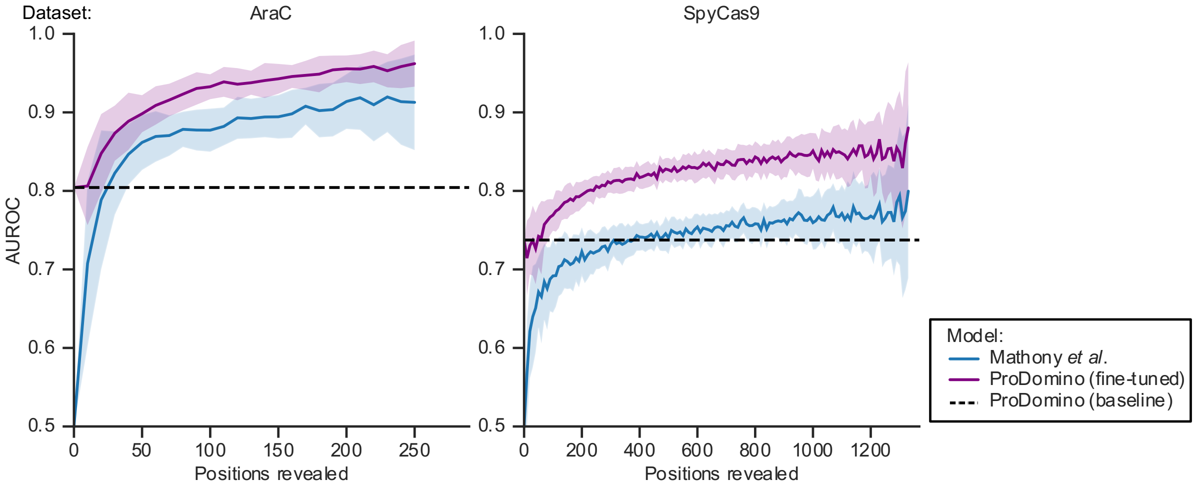

Supplementary Fig. 7 | Benchmarking of ProDomino against a previously published gradient boosting classifier. Gradient boosting classifiers were trained on the indicated number of randomly selected experimental datapoints of AraC and *Spy*Cas9 and performance was tested on the remaining data. Datasets correspond to experimental domain insertion screen by Mathony et al.[1] (AraC) and Oakes et al.[5] (*Spy*Cas9). ProDomino baseline corresponds to the performance of standard ProDomino prior to fine tuning. Purple line indicates the performance of ProDomino when fine-tuned on the number of experimental data points identical to those used for gradient boosting classifier training. Colored shading represents the standard deviation calculated based on 50 trials of randomly selected datasets of the indicated size.

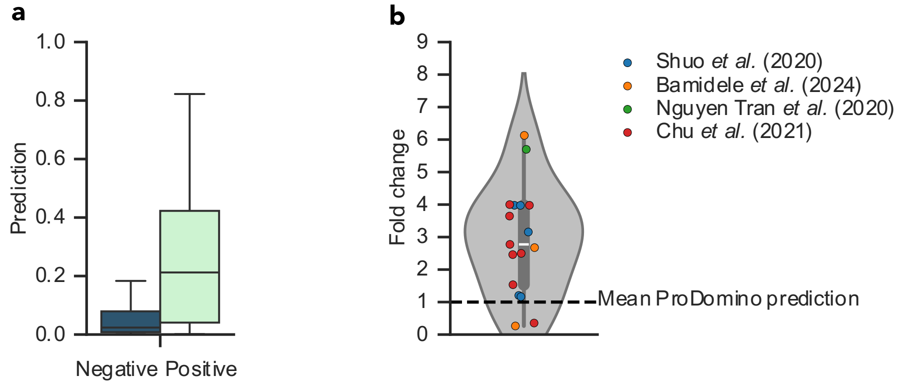

Supplementary Fig. 8 | Cross-validation of ProDomino performance on previously published Datasets. a, Boxplot of ProDomino predictions for insertion sites in Kir2.1 previously published by Coyote-Maestas et al.[2]. The true positive and true negative sites were identified based on Fig. 2 in reference 2 provided they tolerated or did not tolerate at least two of the three inserts tested in the study, respectively. **b**, Insertion sites in different Cas9 orthologues that were successfully employed to construct domain inlaid CRISPR base editors were gathered from the literature[6–9]. The ProDomino-predicted tolerance for domain fusion at the corresponding insertion site, normalized to the mean prediction score for the respective Cas9 ortholog, is shown (i.e. random choice would result in a value of 1).

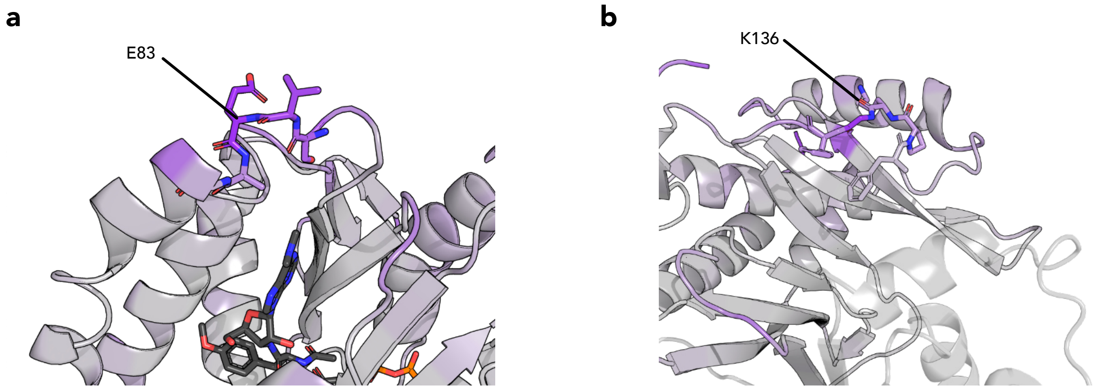

Supplementary Fig. 9 | Detailed view of the insertion sites of the lead PAC and CAT candidates. **a**, **b**, ProDomino inferred insertion scores are mapped onto the crystal structures of PAC (**a**) and CAT (**b**). The insertion sites of the lead candidates are indicated and shown in the side chain representation. PDB ID s: 7K0A, 1PD5.

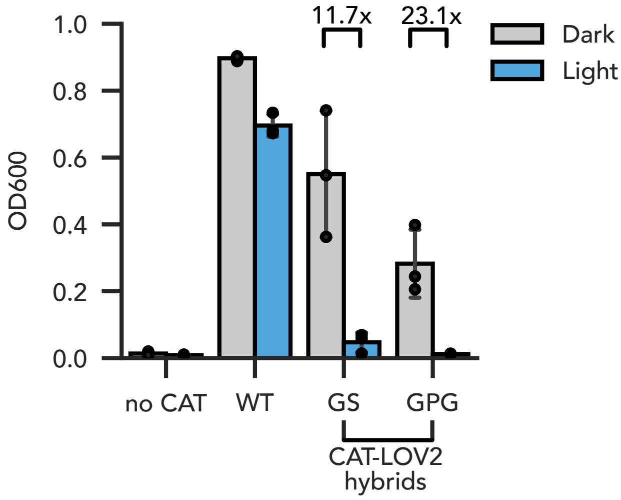

Supplementary Fig. 10 | Light-controlled *E. coli* cell growth. Bacteria were transformed with plasmids expressing the indicated CAT variant (insertion behind K136) or an empty control plasmid. Liquid cultures were grown in the presence of 50 µg/mL chloramphenicol for 7 hours and cell density was assessed by measuring OD at 600 nm. Bars indicate means, error bars the standard deviation, and black dots individual data points from n=3 independent experiments. Amino acid sequences of the symmetric linkers at the receptor domain boundaries are indicated. GS, Glycine-serine linker; GPG, Glycine-proline-serine linker.

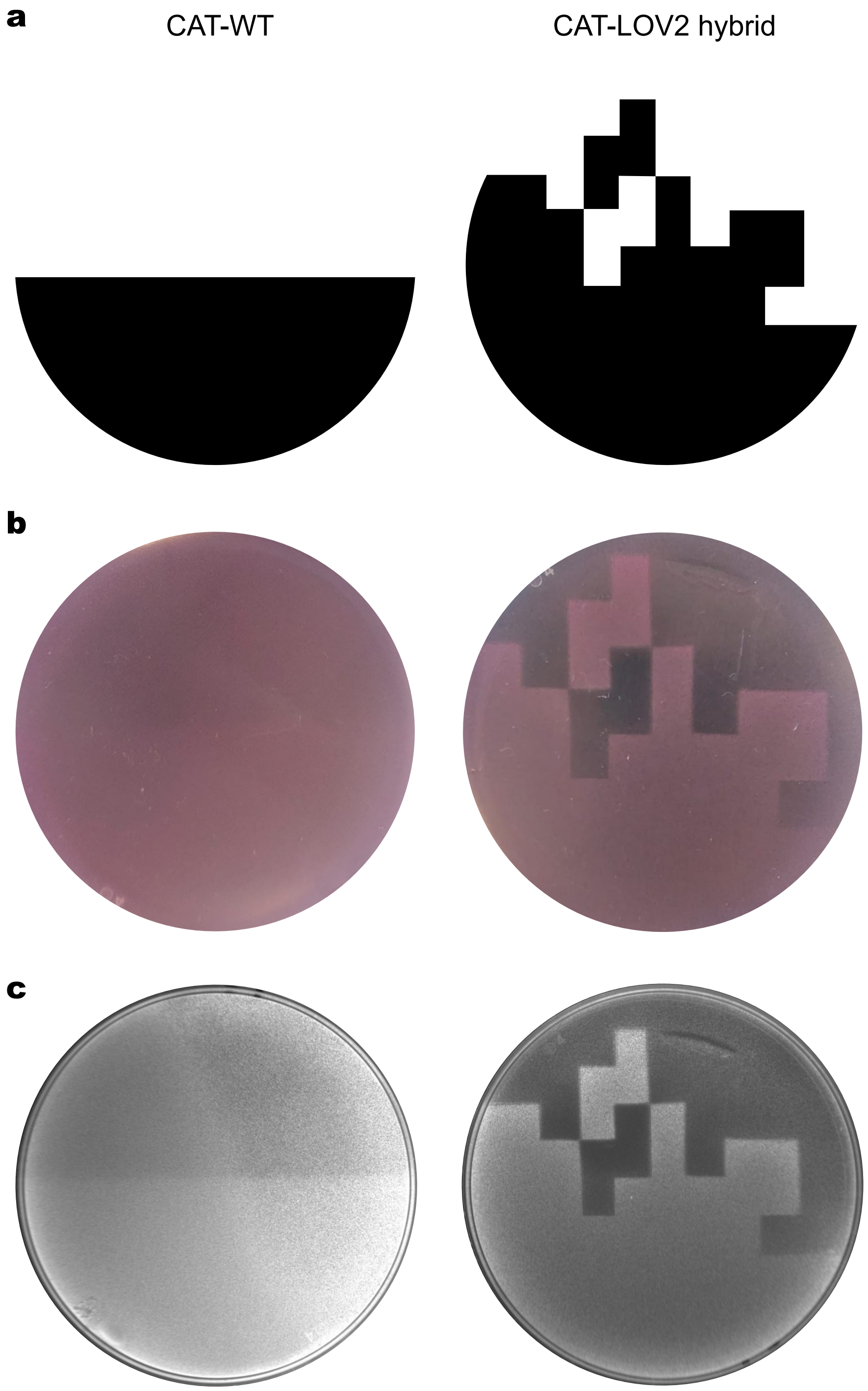

Supplementary Fig. 11 | Spatial control of *E. coli* growth. a-c, *E. coli* expressing CAT-K136-LOV or a constitutively active wild-type CAT and mRFP (for visualization) were plated in top agar supplemented with 25 µg/mL chloramphenicol. During incubation at 37°C, the plates were illuminated through the depicted photomask (**a**) and mRFP was imaged under white light (**b**) or UV light (**c**). The lower right image corresponds to Fig. 3h.

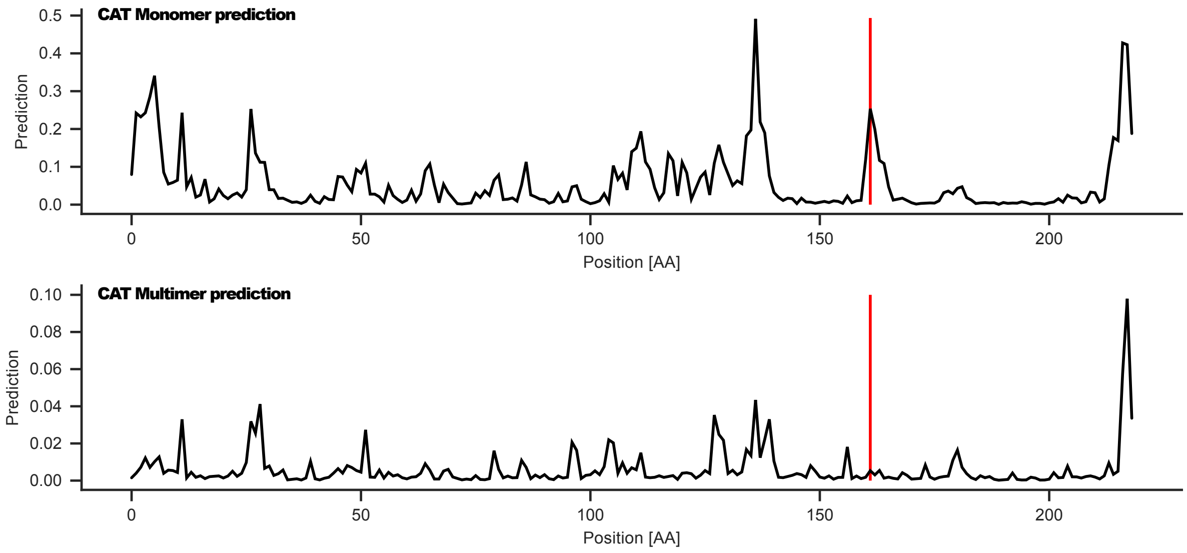

Supplementary Fig. 12 | Insertion score prediction for an artificially concatenated, trimeric CAT correctly identifies a false positive site. ProDomino predictions for CAT are shown based on the wild-type sequence (top panel) or a concatenated 3xCAT sequence (bottom panel) as input. The red line marks the “false positive” site, which we observed not to tolerate PDZ insertion (see Extended Data Fig. 3d).

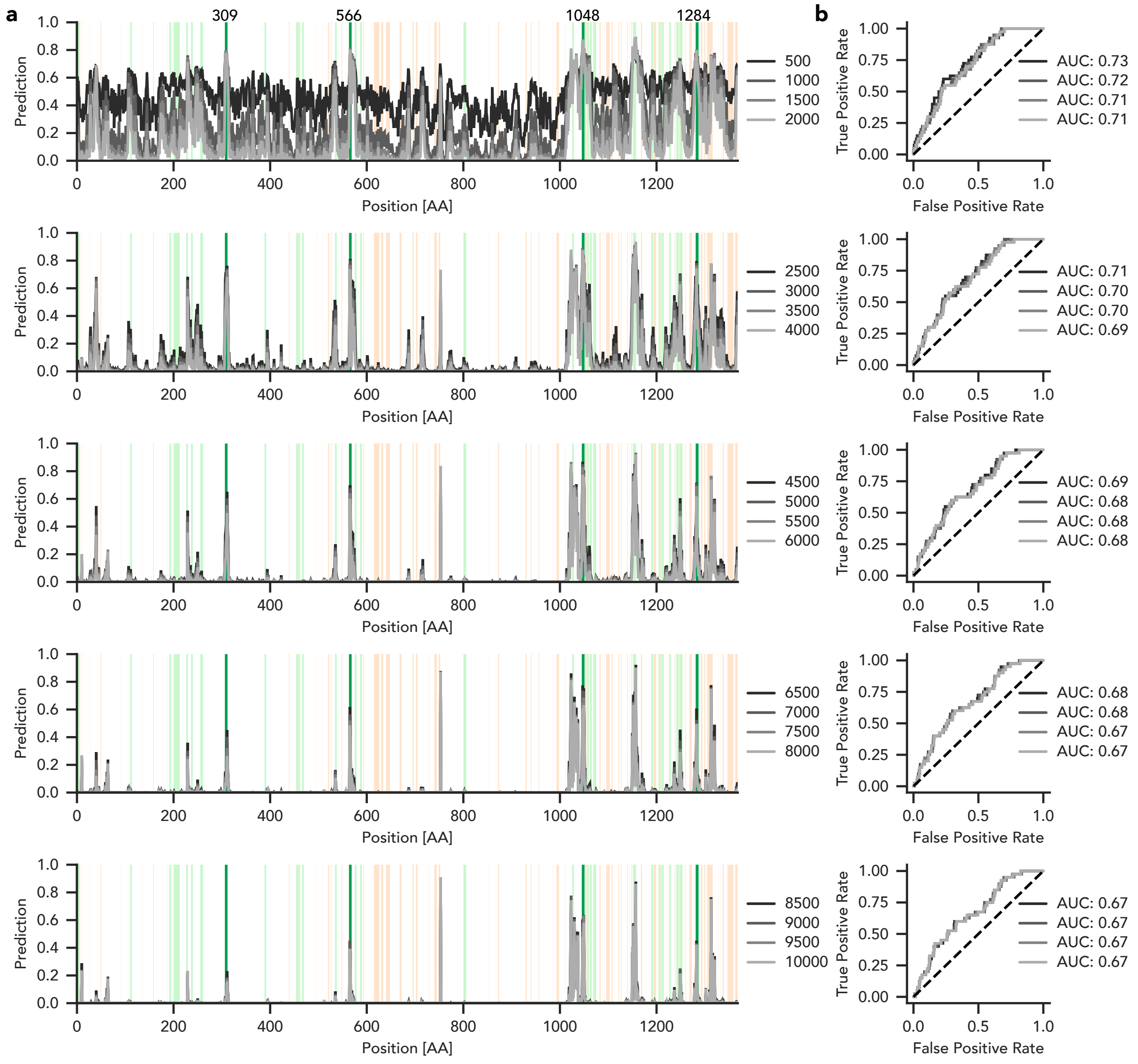

Supplementary Fig. 13 | ProDomino predictions correlate with insertion tolerant regions in Cas9. a, The insertion score for Cas9 is shown for each amino acid position. The scores of models trained for different numbers of steps are shown in different shades of gray. Regions in light green indicate insertion permissive sites, orange marks sites that were not amenable to domain fusion, as reported by Oakes et al.[5]. Positions that have been experimentally validated by us (Fig. 4c) are marked in dark green. **b**, ROC curves based on the predictions in **a** are shown. The area under the curve (AUC) is given for each model.

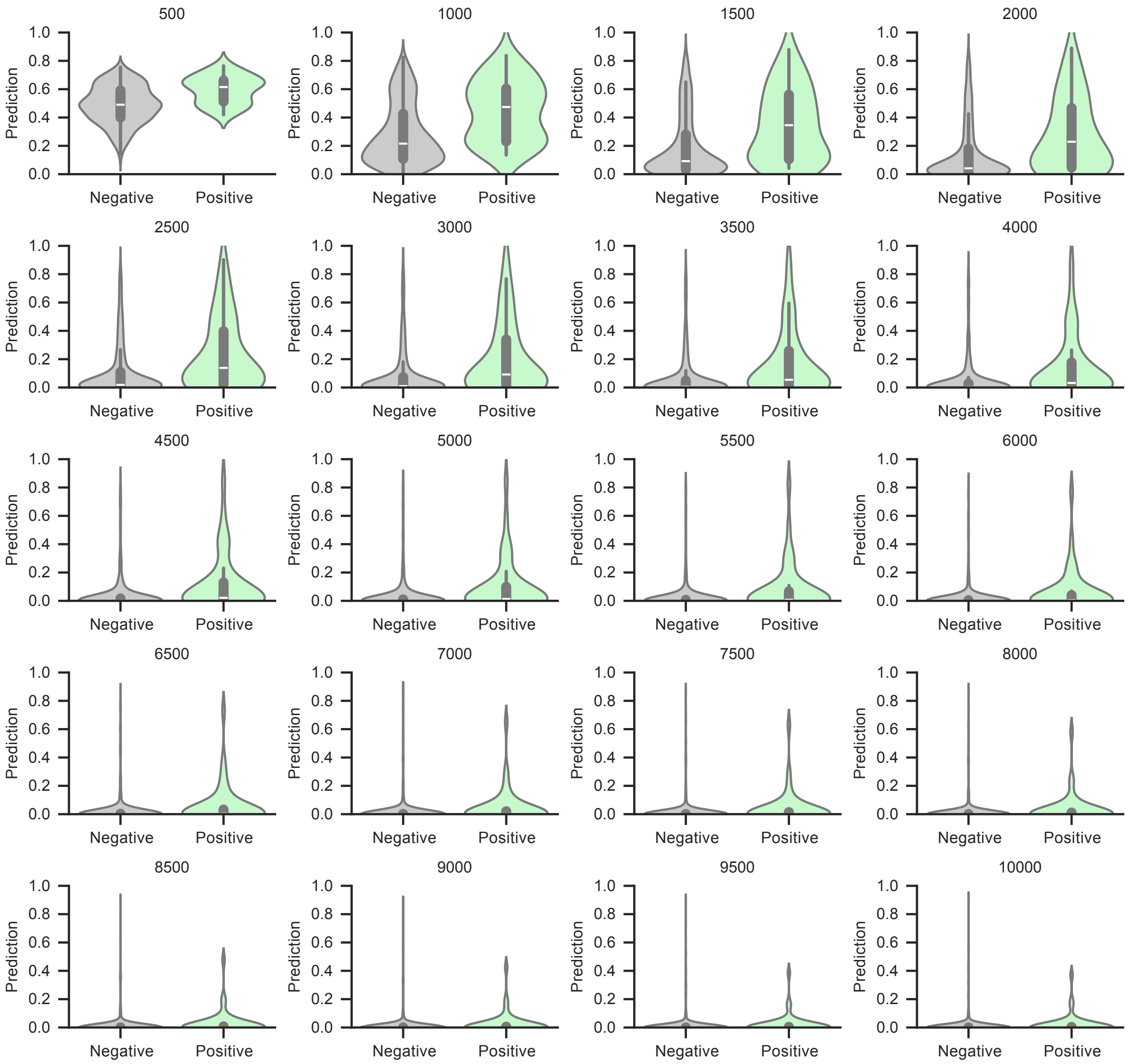

Supplementary Fig. 14 | Comparison of prediction score distributions for insertion permissive or non-permissive sites in Cas9. Violin plots showing the distribution of ProDomino predictions for insertion permissive sites (green) and non-permissive sites (gray). Positive and negative labels correspond to the data reported by Oakes et al.[5]. Boxes represent the interquartile range (IQR) and the median is represented by a white horizontal line. Whiskers extend to the 1.5-fold IQR or to the value of the smallest or largest prediction value.

a

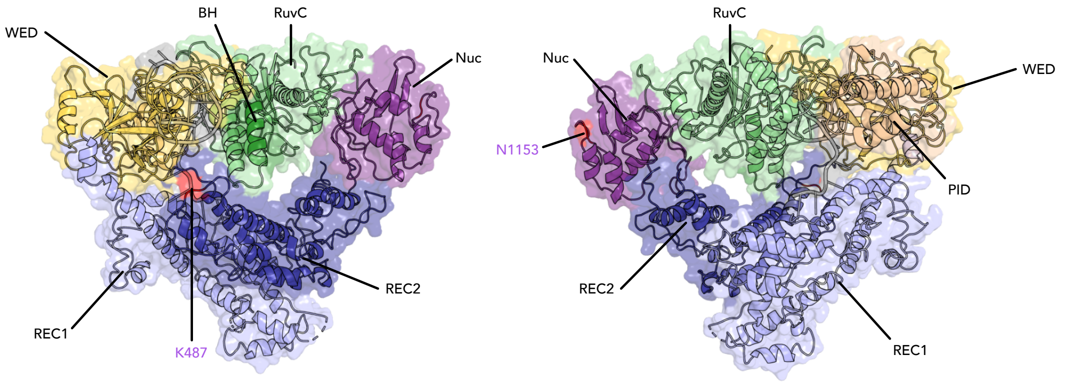

Supplementary Fig. 15 | Positions of the two lead insertion sites within Cas12a. The cryo-EM structure of Cas12a is shown in two orientations. The two allosteric insertion sites, K487 and N1153, are marked in red. The individual domains of Cas12a are indicated and color-coded. WED: wedge; BH: bridge helix; Nuc: nuclease; REC: recognition; PID: PAM interacting domain. PDB ID: 6IV6.

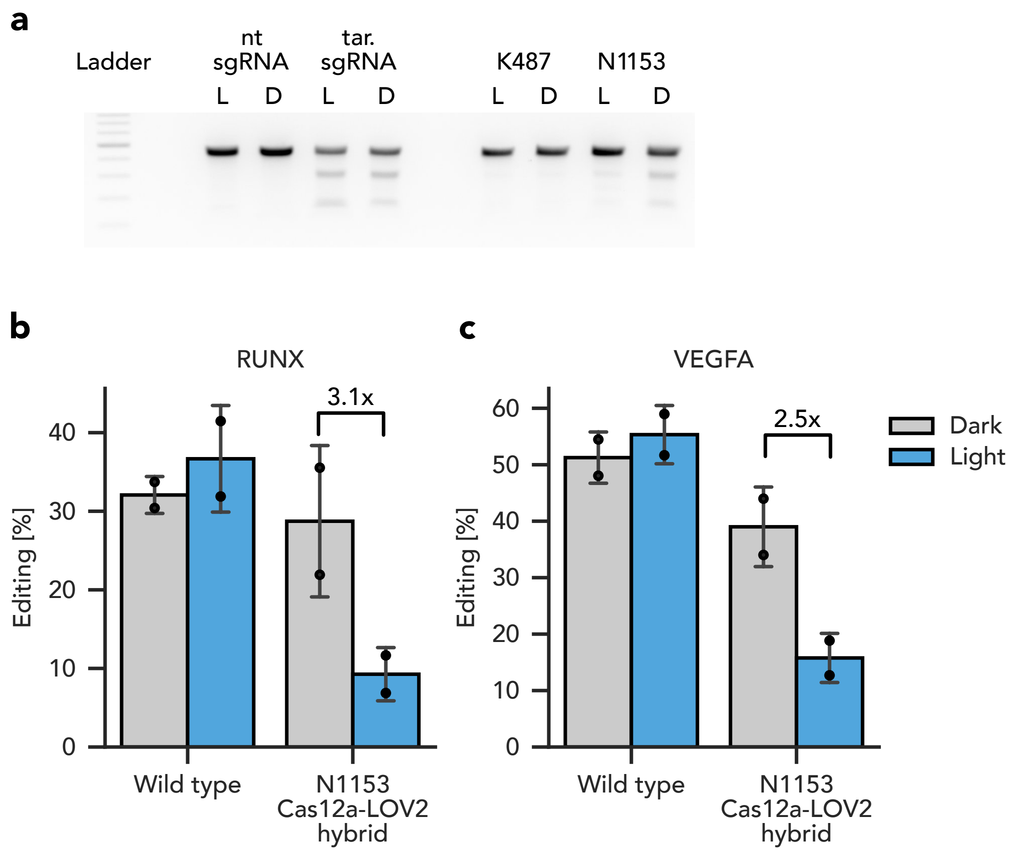

Supplementary Fig. 16 | Light-dependent regulation of *Mb*Cas12a.

a-c, HEK293T cells were transfected with vectors encoding (i) the indicated Cas12a variant and (ii) a gRNA targeting the endogenous *RUNX* (**a**, **b**) or *VEGFA* (**c**) locus. Samples were incubated under blue light illumination or in the dark for 72 h. InDel frequencies were assessed qualitatively via T7E1-assay (**a**) or quantitatively by NGS (**b**, **c**). A representative agarose gel image is shown in **a**. (**b,c**) Bars indicate means, error bars the standard deviation, and black dots individual data points from n=2 independent experiments.

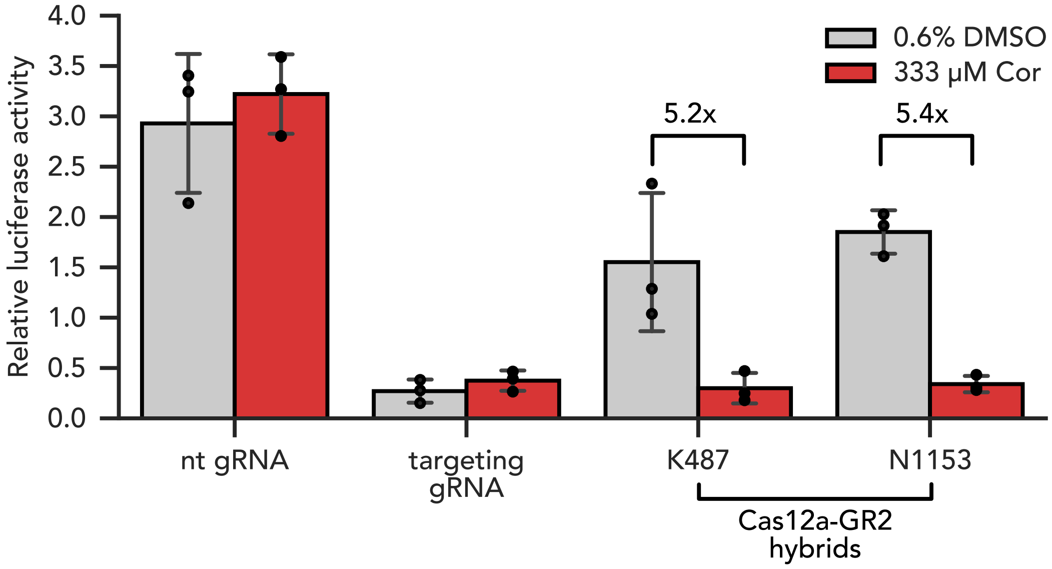

Supplementary Fig. 17 | Tight, chemical regulation of Cas12a. HEK293T cells were transfected with vectors encoding (i) the indicated Cas12a variant, (ii) a firefly luciferase gene targeting gRNA and (iii) a dual luciferase reporter. The Cas12a hybrids carried the GR2 receptor domain as insert at positions K487 or N1153. Samples were treated with cortisol or DMSO (solvent) as indicated. At 48 hours post-transfection, luciferase activity was measured in a plate reader. Bars indicate means, error bars the standard deviation, and black dots individual data points from n=3 independent experiments. Cor, cortisol.

Supplementary Table 1 | List of constructs used in this study. Insert domains are flanked by SG linkers on both sides, unless otherwise indicated. WT, wild type; CMV, cytomegalovirus; eGFP, enhanced green fluorescent protein.

#	Name	Description, sequential order
**1**	BLA_CAT	β-lactamase expression cassette, chloramphenicol acetyltransferase expression cassette
**2**	BLA	β-lactamase expression cassette
**3**	BLA_CAT_F25_PDZ	Chloramphenicol acetyltransferase with a PDZ insertion behind F25
**4**	BLA_CAT_Q26_PDZ	Chloramphenicol acetyltransferase with a PDZ insertion behind Q26
**5**	BLA_CAT_S27_PDZ	Chloramphenicol acetyltransferase with a PDZ insertion behind S27
**6**	BLA_CAT_T36_PDZ	Chloramphenicol acetyltransferase with a PDZ insertion behind T36
**7**	BLA_CAT_F73_PDZ	Chloramphenicol acetyltransferase with a PDZ insertion behind F73
**8**	BLA_CAT_C91_PDZ	Chloramphenicol acetyltransferase with a PDZ insertion behind C91
**9**	BLA_CAT_P135_PDZ	Chloramphenicol acetyltransferase with a PDZ insertion behind P135
**10**	BLA_CAT_K136_PDZ	Chloramphenicol acetyltransferase with a PDZ insertion behind K136
**11**	BLA_CAT_G137_PDZ	Chloramphenicol acetyltransferase with a PDZ insertion behind G137
**12**	BLA_CAT_W150_PDZ	Chloramphenicol acetyltransferase with a PDZ insertion behind W150
**13**	BLA_CAT_V160_PDZ	Chloramphenicol acetyltransferase with a PDZ insertion behind V160
**14**	BLA_CAT_A161_PDZ	Chloramphenicol acetyltransferase with a PDZ insertion behind A161
**15**	BLA_CAT_N162_PDZ	Chloramphenicol acetyltransferase with a PDZ insertion behind N162
**16**	BLA_CAT_D181_PDZ	Chloramphenicol acetyltransferase with a PDZ insertion behind D181
**17**	BLA_CAT_Q190_PDZ	Chloramphenicol acetyltransferase with a PDZ insertion behind Q190
**18**	BLA_CAT_F25_LOV	Chloramphenicol acetyltransferase with a AsLOV2 insertion behind F25
**19**	BLA_CAT_Q26_LOV	Chloramphenicol acetyltransferase with a AsLOV2 insertion behind Q26
**20**	BLA_CAT_S27_LOV	Chloramphenicol acetyltransferase with a AsLOV2 insertion behind S27
**21**	BLA_CAT_T36_LOV	Chloramphenicol acetyltransferase with a AsLOV2 insertion behind T36
**22**	BLA_CAT_F73_LOV	Chloramphenicol acetyltransferase with a AsLOV2 insertion behind F73
**23**	BLA_CAT_C91_LOV	Chloramphenicol acetyltransferase with a AsLOV2 insertion behind C91
**24**	BLA_CAT_P135_LOV	Chloramphenicol acetyltransferase with a AsLOV2 insertion behind P135
**25**	BLA_CAT_K136_LOV	Chloramphenicol acetyltransferase with a AsLOV2 insertion behind K136
**26**	BLA_CAT_G137_LOV	Chloramphenicol acetyltransferase with a AsLOV2 insertion behind G137
**27**	BLA_CAT_W150_LOV	Chloramphenicol acetyltransferase with a AsLOV2 insertion behind W150
**28**	BLA_CAT_V160_LOV	Chloramphenicol acetyltransferase with a AsLOV2 insertion behind V160
**29**	BLA_CAT_A161_LOV	Chloramphenicol acetyltransferase with a AsLOV2 insertion behind A161
**30**	BLA_CAT_N162_LOV	Chloramphenicol acetyltransferase with a AsLOV2 insertion behind N162
**31**	BLA_CAT_D181_LOV	Chloramphenicol acetyltransferase with a AsLOV2 insertion behind D181
**32**	BLA_CAT_Q190_LOV	Chloramphenicol acetyltransferase with a AsLOV2 insertion behind Q190
**33**	BLA_CAT_K136_GPG_LOV2	Chloramphenicol acetyltransferase with a AsLOV2 insertion behind K136, flanked by GPG linkers
**34**	eGFP	CMV promoter, eGFP, bGH-polyA
**35**	PAC_eGFP	CMV promoter, puromycin acetyltransferase, T2A sequence, eGFP, bGH-polyA
**36**	PAC_D29_PDZ_eGFP	CMV promoter, puromycin acetyltransferase with a PDZ insertion behind D29, T2A sequence, eGFP, bGH-polyA
**37**	PAC_G58_PDZ_eGFP	CMV promoter, puromycin acetyltransferase with a PDZ insertion behind G58, T2A sequence, eGFP, bGH-polyA
**38**	PAC_V75_PDZ_eGFP	CMV promoter, puromycin acetyltransferase with a PDZ insertion behind V75, T2A sequence, eGFP, bGH-polyA
**39**	PAC_S81_PDZ_eGFP	CMV promoter, puromycin acetyltransferase with a PDZ insertion behind S81, T2A sequence, eGFP, bGH-polyA
**40**	PAC_V82_PDZ_eGFP	CMV promoter, puromycin acetyltransferase with a PDZ insertion behind V82, T2A sequence, eGFP, bGH-polyA
**41**	PAC_E83_PDZ_eGFP	CMV promoter, puromycin acetyltransferase with a PDZ insertion behind E83, T2A sequence, eGFP, bGH-polyA
**42**	PAC_A84_PDZ_eGFP	CMV promoter, puromycin acetyltransferase with a PDZ insertion behind A84, T2A sequence, eGFP, bGH-polyA
**43**	PAC_G85_PDZ_eGFP	CMV promoter, puromycin acetyltransferase with a PDZ insertion behind G85, T2A sequence, eGFP, bGH-polyA
**44**	PAC_A86_PDZ_eGFP	CMV promoter, puromycin acetyltransferase with a PDZ insertion behind A86, T2A sequence, eGFP, bGH-polyA
**45**	PAC_S101_PDZ_eGFP	CMV promoter, puromycin acetyltransferase with a PDZ insertion behind S101, T2A sequence, eGFP, bGH-polyA
**46**	PAC_G111_PDZ_eGFP	CMV promoter, puromycin acetyltransferase with a PDZ insertion behind G111, T2A sequence, eGFP, bGH-polyA
**47**	PAC_K119_PDZ_eGFP	CMV promoter, puromycin acetyltransferase with a PDZ insertion behind K119, T2A sequence, eGFP, bGH-polyA
**48**	PAC_E120_PDZ_eGFP	CMV promoter, puromycin acetyltransferase with a PDZ insertion behind E120, T2A sequence, eGFP, bGH-polyA
**49**	PAC_P121_PDZ_eGFP	CMV promoter, puromycin acetyltransferase with a PDZ insertion behind P121, T2A sequence, eGFP, bGH-polyA

**50**	PAC_P185_PDZ_eGFP	CMV promoter, puromycin acetyltransferase with a PDZ insertion behind P185, T2A sequence, eGFP, bGH-polyA
**51**	PAC_E83_LOV_eGFP	CMV promoter, puromycin acetyltransferase with a AsLOV2 insertion behind E83, T2A sequence, eGFP, bGH-polyA
**52**	Renilla luciferase	HSV TK promoter, Renilla luciferase, SV40 polyA (Promega)
**53**	TetO_firefly_luciferase[10]	TetO, firefly luciferase
**54**	sgRNA-Tet_RLuc (SpCas9)[11]	U6 promoter, TetO-targeting sgRNA for MbCas12a, polyT; HSV TK promoter, Renilla luciferase, SV40 polyA
**55**	d*Sp*Cas9-VPR	EF-1α promoter, SV40 NLS, d*Sp*Cas9, SV40 NLS, VPR, beta-globin polyA
**56**	d*Sp*Cas9-N309-LOV-VPR	EF-1α promoter, SV40 NLS, d*Sp*Cas9 with an AsLOV2 insertion behind N309, SV40 NLS, VPR, beta-globin polyA
**57**	d*Sp*Cas9-E566-LOV-VPR	EF-1α promoter, SV40 NLS, d*Sp*Cas9 with an AsLOV2 insertion behind E566, SV40 NLS, VPR, beta-globin polyA
**58**	d*Sp*Cas9-T1048-LOV-VPR	EF-1α promoter, SV40 NLS, d*Sp*Cas9 with an AsLOV2 insertion behind T1048, SV40 NLS, VPR, beta-globin polyA
**59**	d*Sp*Cas9-D1284-LOV-VPR	EF-1α promoter, SV40 NLS, d*Sp*Cas9 with an AsLOV2 insertion behind D1284, SV40 NLS, VPR, beta-globin polyA
**60**	Firefly luciferase[12]	TK promoter, firefly luciferase
**61**	nt-sgRNA (*Mb*Cas12a)	U6 promoter, non-targeting sgRNA for MbCas12a, polyT
**62**	ff-sgRNA (*Mb*Cas12a)	U6 promoter, firefly luciferase-targeting sgRNA for MbCas12a, polyT
**63**	MbCas12a[13]	CMV promoter, MbCas12a, nucleoplasmin NLS, beta-globin polyA
**64**	MbCas12a_F35_PDZ	CMV promoter, MbCas12a with a PDZ insertion behind F35, nucleoplasmin NLS, beta-globin polyA
**65**	MbCas12a_L36_PDZ	CMV promoter, MbCas12a with a PDZ insertion behind L36, nucleoplasmin NLS, beta-globin polyA
**66**	MbCas12a_N37_PDZ	CMV promoter, MbCas12a with a PDZ insertion behind N37, nucleoplasmin NLS, beta-globin polyA
**67**	MbCas12a_T71_PDZ	CMV promoter, MbCas12a with a PDZ insertion behind T71, nucleoplasmin NLS, beta-globin polyA
**68**	MbCas12a_K72_PDZ	CMV promoter, MbCas12a with a PDZ insertion behind K72, nucleoplasmin NLS, beta-globin polyA
**69**	MbCas12a_L73_PDZ	CMV promoter, MbCas12a with a PDZ insertion behind L73, nucleoplasmin NLS, beta-globin polyA
**70**	MbCas12a_N113_PDZ	CMV promoter, MbCas12a with a PDZ insertion behind N113, nucleoplasmin NLS, beta-globin polyA
**71**	MbCas12a_G114_PDZ	CMV promoter, MbCas12a with a PDZ insertion behind G114, nucleoplasmin NLS, beta-globin polyA
**72**	MbCas12a_G115_PDZ	CMV promoter, MbCas12a with a PDZ insertion behind G115, nucleoplasmin NLS, beta-globin polyA
**73**	MbCas12a_I486_PDZ	CMV promoter, MbCas12a with a PDZ insertion behind I486, nucleoplasmin NLS, beta-globin polyA
**74**	MbCas12a_K487_PDZ	CMV promoter, MbCas12a with a PDZ insertion behind K487, nucleoplasmin NLS, beta-globin polyA
**75**	MbCas12a_S488_PDZ	CMV promoter, MbCas12a with a PDZ insertion behind S488, nucleoplasmin NLS, beta-globin polyA
**76**	MbCas12a_P1152_PDZ	CMV promoter, MbCas12a with a PDZ insertion behind P1152, nucleoplasmin NLS, beta-globin polyA
**77**	MbCas12a_N1153_PDZ	CMV promoter, MbCas12a with a PDZ insertion behind N1153, nucleoplasmin NLS, beta-globin polyA
**78**	MbCas12a_L1154_PDZ	CMV promoter, MbCas12a with a PDZ insertion behind L1154, nucleoplasmin NLS, beta-globin polyA
**79**	MbCas12a_D55_PDZ	CMV promoter, MbCas12a with a PDZ insertion behind D55, nucleoplasmin NLS, beta-globin polyA
**80**	MbCas12a_K105_PDZ	CMV promoter, MbCas12a with a PDZ insertion behind K105, nucleoplasmin NLS, beta-globin polyA
**81**	MbCas12a_I426_PDZ	CMV promoter, MbCas12a with a PDZ insertion behind I426, nucleoplasmin NLS, beta-globin polyA
**82**	MbCas12a_L501_PDZ	CMV promoter, MbCas12a with a PDZ insertion behind L501, nucleoplasmin NLS, beta-globin polyA
**83**	MbCas12a_A802_PDZ	CMV promoter, MbCas12a with a PDZ insertion behind A802, nucleoplasmin NLS, beta-globin polyA
**84**	MbCas12a_K487_GP_AsLOV2	CMV promoter, MbCas12a with a AsLOV2 insertion behind K487 flanked by a GP linker, nucleoplasmin NLS, beta-globin polyA
**85**	MbCas12a_N1153_GP_AsLOV2	CMV promoter, MbCas12a with a AsLOV2 insertion behind N1153 flanked by a GP linker, nucleoplasmin NLS, beta-globin polyA
**86**	MbCas12a_K487_GPG_GR2	CMV promoter, MbCas12a with a cpGR2 insertion behind K487 flanked by a GPPG linker, nucleoplasmin NLS, beta-globin polyA
**87**	MbCas12a_N1153_GPG_GR2	CMV promoter, MbCas12a with a cpGR2 insertion behind N1153 flanked by a GPPG linker, nucleoplasmin NLS, beta-globin polyA
**88**	VEGFA-sgRNA (*Mb*Cas12a)	U6 promoter, VEGFA targeting sgRNA for MbCas12a, polyT
**89**	RUNX-sgRNA (*Mb*Cas12a)	U6 promoter, RUNX targeting sgRNA for MbCas12a, polyT
**90**	GRIN2B-sgRNA (*Mb*Cas12a)	U6 promoter, GRIN2B targeting sgRNA for MbCas12a, polyT

Supplementary Table 2 | Amino acid sequences of the domains and proteins used in this study. Blue: linker sequences; orange: affinity tag; red: T2A sequence; green: nuclear localization sequence.

Protein / Domain	Amino acid sequence
Chloramphenicol acetyltransferase	MEKKITGYTTVDISQWHRKEHFEAFQSVAQCTYNQTVQLDITAFLKTVKKNKHKFY<br>PAFIHILARLMNAHPEFRMAMKDGELVIWDSVHPCYTVFHEQTETFSSLLSEYHDD<br>FRQFLHIYSQDVACYGENLAYFPKGFIENMFFVSANPWVSFTSFDLNVANMDNFF<br>APVFTMGKYYTQGDKVLMPLAIQVHHAVCDGFHVGRMLNELQQYCDEWQGGA
*As*LOV2	SGLATTLERIEKNFVITDPRLPDNPIIFASDSFLQLTEYSREEILGRNCRFLQGPETD<br>RATVRKIRDAIDNQTEVTVQLINYTKSGKKFWNLFHLQPMRDQKGDVQYFIGVQLD<br>GTEHVRDAAEREGVMLIKKTAENIDEAAKGS
PDZ	SGRRRVTVRKADAGGLGISIKGGRENKMPILISKIFKGLAADQTEALFVGDAILSVN<br>GEDLSSATHDEAVQALKKTGKEVVLEVKYMKGS
GR2	GPGNSSQNWQRFYQLTKLLDSMHEMVGGLLQFCFYTFVNKSLSVEFPEMLAEIIS<br>NQLPKFKAGSVKPLLFHQKGGGSGGSGGSGGSGGSGLISLLEVIEPEVLYSGYDS<br>TLPDTSTRLMSTLNRLGGRQVVSAVKWAKALPGFRNLHLDDQMTLLQYSWMSLM<br>AFSLGWRSYKQSNGNMLCFAPDLVINEERMQLPYMYDQCQQMLKISSEFVRLQV<br>SYDEYLCMKVLLLLSTVPKDGLKSQAVFDEIRMTYIKELGKAIVKREGGPG
Puromycin acetyltransferase-T2A-eGFP	MTEYKPTVRLATRDDVPRAVRTLAAAFADYPATRHTVDPDRHIERVTELQELFLTR<br>VGLDIGKVWVADDGAAVAVWTTPESVEAGAVFAEIGPRMAELSGSRLAAQQQME<br>GLLAPHRPKEPAWFLATVGVSPDHQGKGLGSAVVLPGVEAAERAGVPAFLETSA<br>PRNLPFYERLGFTVTADVEVPEGPRTWCMTRKPGAGSTGSRGSGEGRGSLLTC<br>GDVEENPGPMVSKGEELFTGVVPILVELDGDVNGHKFSVSGEGEGDATYGKLTLK<br>FICTTGKLPVPWPTLVTTLTYGVQCFSRYPDHMKQHDFFKSAMPEGYVQERTIFF<br>KDDGNYKTRAEVKFEGDTLVNRIELKGIDFKEDGNILGHKLEYNYNSHNVYIMADK<br>QKNGIKVNFKIRHNIEDGSVQLADHYQQNTPIGDGPVLLPDNHYLSTQSALSKDPN<br>EKRDHMVLLEFVTAAGITLGMDELYK
*Mb*Cas12a	MLFQDFTHLYPLSKTVRFELKPIGKTLEHIHAKNFLNQDETMADMYQKVKAILDDY<br>HRDFIADMMGEVKLTKLAEFYDVYLKFRKNPKDDGLQKQLKDLQAVLRKEIVKPIG<br>NGGKYKAGYDRLFGAKLFKDGKELGDLAKFVIAQEGESSPKLAHLAHFEKFSTYFT<br>GFHDNRKNMYSDEDKHTAIAYRLIHENLPRFIDNLQILATIKQKHSALYDQIINELTA<br>SGLDVSLASHLDGYHKLLTQEGITAYNTLLGGISGEAGSRKIQGINELINSHHNQHC<br>HKSERIAKLRPLHKQILSDGMGVSFLPSKFADDSEVCQAVNEFYRHYADVFAKVQ<br>SLFDGFDDYQKDGIYVEYKNLNELSKQAFGDFALLGRVLDGYYVDVVNPEFNERF<br>AKAKTDNAKAKLTKEKDKFIKGVHSLASLEQAIEHYTARHDDESVQAGKLGQYFKH<br>GLAGVDNPIQKIHNNHSTIKGFLERERPAGERALPKIKSDKSPEIRQLKELLDNALN<br>VAHFAKLLTTKTTLHNQDGNFYGEFGALYDELAKIATLYNKVRDYLSQKPFSTEKY<br>KLNFGNPTLLNGWDLNKEKDNFGVILQKDGCYYLALLDKAHKKVFDNAPNTGKSV<br>YQKMIYKLLPGPNKMLPKVFFAKSNLDYYNPSAELLDKYAQGTHKKGDNFNLKDC<br>HALIDFFKAGINKHPEWQHFGFKFSPTSSYQDLSDFYREVEPQGYQVKFVDINADY<br>INELVEQGQLYLFQIYNKDFSPKAHGKPNLHTLYFKALFSEDNLVNPIYKLNGEAEIF<br>YRKASLDMNETTIHRAGEVLENKNPDNPKKRQFVYDIIKDKRYTQDKFMLHVPITM<br>NFGVQGMTIKEFNKKVNQSIQQYDEVNVIGIDRGERHLLYLTVINSKGEILEQRSLN<br>DITTASANGTQMTTPYHKILDKREIERLNARVGWGEIETIKELKSGYLSHVVHQISQ<br>LMLKYNAIVVLEDLNFGFKRGRFKVEKQIYQNFENALIKKLNHLVLKDKADDEIGSY<br>KNALQLTNNFTDLKSIGKQTGFLFYVPAWNTSKIDPETGFVDLLKPRYENIAQSQAF<br>FGKFDKICYNADRGYFEFHIDYAKFNDKAKNSRQIWKICSHGDKRYVYDKTANQN<br>KGATIGVNVNDELKSLFTRYHINDKQPNLVMDICQNNDKEFHKSLMYLLKTLLALRY<br>SNASSDEDFILSPVANDEGVFFNSALADDTQPQNADANGAYHIALKGLWLLNELKN<br>SDDLNKVKLAIDNQTWLNFAQNRKRPAATKKAGQAKKKKGSYPYDVPDYAYPYD<br>VPDYAYPYDVPDYA
d*Sp*Cas9-VPR	MSPKKKRKVEASDKKYSIGLAIGTNSVGWAVITDEYKVPSKKFKVLGNTDRHSIKK<br>NLIGALLFDSGETAEATRLKRTARRRYTRRKNRICYLQEIFSNEMAKVDDSFFHRLE<br>ESFLVEEDKKHERHPIFGNIVDEVAYHEKYPTIYHLRKKLVDSTDKADLRLIYLALAH<br>MIKFRGHFLIEGDLNPDNSDVDKLFIQLVQTYNQLFEENPINASGVDAKAILSARLS<br>KSRRLENLIAQLPGEKKNGLFGNLIALSLGLTPNFKSNFDLAEDAKLQLSKDTYDDD<br>LDNLLAQIGDQYADLFLAAKNLSDAILLSDILRVNTEITKAPLSASMIKRYDEHHQDL<br>TLLKALVRQQLPEKYKEIFFDQSKNGYAGYIDGGASQEEFYKFIKPILEKMDGTEEL<br>LVKLNREDLLRKQRTFDNGSIPHQIHLGELHAILRRQEDFYPFLKDNREKIEKILTFRI<br>PYYVGPLARGNSRFAWMTRKSEETITPWNFEEVVDKGASAQSFIERMTNFDKNLP<br>NEKVLPKHSLLYEYFTVYNELTKVKYVTEGMRKPAFLSGEQKKAIVDLLFKTNRKV<br>TVKQLKEDYFKKIECFDSVEISGVEDRFNASLGTYHDLLKIIKDKDFLDNEENEDILE<br>DIVLTLTLFEDREMIEERLKTYAHLFDDKVMKQLKRRRYTGWGRLSRKLINGIRDK<br>QSGKTILDFLKSDGFANRNFMQLIHDDSLTFKEDIQKAQVSGQGDSLHEHIANLAG

	SPAIKKGILQTVKVVDELVKVMGRHKPENIVIEMARENQTTQKGQKNSRERMKRIE<br>EGIKELGSQILKEHPVENTQLQNEKLYLYYLQNGRDMYVDQELDINRLSDYDVDAI<br>VPQSFLKDDSIDNKVLTRSDKNRGKSDNVPSEEVVKKMKNYWRQLLNAKLITQRK<br>FDNLTKAERGGLSELDKAGFIKRQLVETRQITKHVAQILDSRMNTKYDENDKLIREV<br>KVITLKSKLVSDFRKDFQFYKVREINNYHHAHDAYLNAVVGTALIKKYPKLESEFVY<br>GDYKVYDVRKMIAKSEQEIGKATAKYFFYSNIMNFFKTEITLANGEIRKRPLIETNGE<br>TGEIVWDKGRDFATVRKVLSMPQVNIVKKTEVQTGGFSKESILPKRNSDKLIARKK<br>DWDPKKYGGFDSPTVAYSVLVVAKVEKGKSKKLKSVKELLGITIMERSSFEKNPID<br>FLEAKGYKEVKKDLIIKLPKYSLFELENGRKRMLASAGELQKGNELALPSKYVNFLY<br>LASHYEKLKGSPEDNEQKQLFVEQHKHYLDEIIEQISEFSKRVILADANLDKVLSAY<br>NKHRDKPIREQAENIIHLFTLTNLGAPAAFKYFDTTIDRKRYTSTKEVLDATLIHQSIT<br>GLYETRIDLSQLGGDSAGGGGSGGGGSGGGGSGPKKKRKVAAAGSGRADALDD<br>FDLDMLGSDALDDFDLDMLGSDALDDFDLDMLGSDALDDFDLDMLINTSGGSGG<br>GSGGSSQYLPDTDDRHRIEEKRKRTYETFKSIMKKSPFSGPTDPRPPPRRIAVPSR<br>SSASVPKPAPQPYPFTSSLSTINYDEFPTMVFPSGQISQASALAPAPPQVLPQAPA<br>PAPAPAMVSALAQAPAPVPVLAPGPPQAVAPPAPKPTQAGEGTLSEALLQLQFDD<br>EDLGALLGNSTDPAVFTDLASVDNSEFQQLLNQGIPVAPHTTEPMLMEYPEAITRL<br>VTGAQRPPDPAPAPLGAPGLPNGLLSGDEDFSSIADMDFSALLGSGSGSRDSRE<br>GMFLPKPEAGSAISDVFEGREVCQPKRIRPFHPPGSPWANRPLPASLAPTPTGPV<br>HEPVGSLTPAPVPQPLDPAPAVTPEASHLLEDPDEDTSQAVKALREMADTVIPQK<br>EEAAICGQMDLSHPPPRGHLDELTTTLESMTEDLNLDSPLTPELNEILDTFLNDECL<br>LHAMHISTGLSIFDTSLFG

Supplementary Table 3 | gRNAs used in this study. Spacer sequences are marked in bold, PAM motifs are underlined.

Target	Sequence 5'-3'	Cas protein	Source
RUNX	TTTA**CCTTCGGAGCGAAAACCAAG**	*Mb*Cas12a	[13]
GRIN2B	TTTG**GTGCTCAATGAAAGGAGATAAGG**	*Mb*Cas12a	[13]
VEGFA	TTTG**CTAGGAATATTGAAGGGGGC**	*Mb*Cas12a	[14]
Firefly luciferase	TTTC**GGCAACCAGATCATCCCCGACAC**	*Mb*Cas12a	This work
TetO	**TCTCTATCACTGATAGGGAG**TGG	*Sp*Cas9	[11]

Supplementary Table 4 | Primers for amplification of genomic loci.

Gene	Orientation	Primer sequence 5'-3'
RUNX	Forward	CCAGAGGTATCCAGCAGAGG
	Reverse	TACAGGCAAAGCTGAGCAAA
GRIN2B	Forward	GTGTATGCATACTCGCATGGC
	Reverse	CAGGACGGCCAACACCAAC
VEGFA	Forward	GGGTCACTCCAGGATTCCAATAG
	Reverse	GCAATGAAGGGGAAGCTCGAC

Supplementary references

1. Mathony, J., Aschenbrenner, S., Becker, P. & Niopek, D. Dissecting the Determinants of Domain Insertion Tolerance and Allostery in Proteins. *Adv. Sci.* **10**, 2303496 (2023).
2. Coyote-Maestas, W., He, Y., Myers, C. L. & Schmidt, D. Domain insertion permissibility-guided engineering of allostery in ion channels. *Nat. Commun.* **10**, 1–14 (2019).
3. Lin, Z. *et al.* Evolutionary-scale prediction of atomic-level protein structure with a language model. *Science* **379**, 1123–1130 (2023).
4. Devlin, J., Chang, M.-W., Lee, K. & Toutanova, K. BERT: Pre-training of Deep Bidirectional Transformers for Language Understanding. Preprint at http://arxiv.org/abs/1810.04805 (2019).
5. Oakes, B. L. *et al.* Profiling of engineering hotspots identifies an allosteric CRISPR-Cas9 switch. *Nat. Biotechnol.* **34**, 646–651 (2016).
6. Bamidele, N. *et al.* Domain-inlaid Nme2Cas9 adenine base editors with improved activity and targeting scope. *Nat. Commun.* **15**, 1458 (2024).
7. Thuan, M. *et al.* Engineering domain-inlaid SaCas9 adenine base editors with reduced RNA off-targets and increased on-target DNA editing. *Nat. Commun.* **11**, 4871 (2020).
8. Li, S. *et al.* Docking sites inside Cas9 for adenine base editing diversification and RNA off-target elimination. *Nat. Commun.* **11**, 5827 (2020).
9. Chu, S.H. *et al*. Rationally Designed Base Editors for Precise Editing of the Sickle Cell Disease Mutation. *CRISPR J*. **4**, 169-177 (2021).
10. Nihongaki, Y., Yamamoto, S., Kawano, F., Suzuki, H. & Sato, M. CRISPR-Cas9-based Photoactivatable Transcription System. *Chem. Biol.* **22**, 169–174 (2015).
11. Muench, P. *et al.* A modular toolbox for the optogenetic deactivation of transcription. *Nucleic Acids Res.* gkae1237 (2024).
12. Bubeck, F. *et al.* Engineered anti-CRISPR proteins for optogenetic control of CRISPR–Cas9. *Nat. Methods* **15**, 924–927 (2018).
13. Kleinstiver, B. P. *et al.* Discovery of widespread type I and type V CRISPR-Cas inhibitors. *Science* **362**, 240–242 (2018).
14. Kweon, J. *et al.* Fusion guide RNAs for orthogonal gene manipulation with Cas9 and Cpf1. *Nat. Commun.* **8**, 1723 (2017).
