# Supplementary material for: Rational engineering of allosteric protein switches by in silico prediction of domain insertion sites
Source: Nat Methods. 2025 Aug 4;22(8):1698–706. doi: 10.1038/s41592-025-02741-z (PMC12328240; doi:10.1038/s41592-025-02741-z)
Supplement: Supplementary file 2 — Reporting Summary [file 41592_2025_2741_MOESM2_ESM.pdf]

nature portfolio

Corresponding author(s): Jan Mathony, Dominik Niopek

Last updated by author(s): Apr 8, 2025

Reporting Summary

Nature Portfolio wishes to improve the reproducibility of the work that we publish. This form provides structure for consistency and transparency in reporting. For further information on Nature Portfolio policies, see our Editorial Policies and the Editorial Policy Checklist.

Statistics

For all statistical analyses, confirm that the following items are present in the figure legend, table legend, main text, or Methods section.

n/a	Confirmed	
☐	☒	The exact sample size (*n*) for each experimental group/condition, given as a discrete number and unit of measurement
☐	☒	A statement on whether measurements were taken from distinct samples or whether the same sample was measured repeatedly
☒	☐	The statistical test(s) used AND whether they are one- or two-sided<br>*Only common tests should be described solely by name; describe more complex techniques in the Methods section.*
☒	☐	A description of all covariates tested
☒	☐	A description of any assumptions or corrections, such as tests of normality and adjustment for multiple comparisons
☐	☒	A full description of the statistical parameters including central tendency (e.g. means) or other basic estimates (e.g. regression coefficient) AND variation (e.g. standard deviation) or associated estimates of uncertainty (e.g. confidence intervals)
☒	☐	For null hypothesis testing, the test statistic (e.g. *F*, *t*, *r*) with confidence intervals, effect sizes, degrees of freedom and *P* value noted<br>*Give P values as exact values whenever suitable.*
☒	☐	For Bayesian analysis, information on the choice of priors and Markov chain Monte Carlo settings
☒	☐	For hierarchical and complex designs, identification of the appropriate level for tests and full reporting of outcomes
☒	☐	Estimates of effect sizes (e.g. Cohen's *d*, Pearson's *r*), indicating how they were calculated

Our web collection on statistics for biologists contains articles on many of the points above.

Software and code

Policy information about availability of computer code

Data collection	Luciferase data, fluorescence, absorbance and optical density data were obtained using the i-control software (Tecan, version 2.0). Microscopy images were acquired and processed with the Keyence BZ-II Viewer (Keyence, version 1.01) and BZ-II Analyzer software (Keyence, version 1.01). Our computational pipeline is based on the Uniref50 dataset (June 28, 2023) and the CATH database (June 29, 2023)
Data analysis	Data anaylsis was performed in Python 3.1 using numpy 1.26, biopython 1.81, seaborn 0.13.1, matplotlib 3.8.1, py3Dmol 2.1 and notepook 7.0.6. Machine learning models were traned using pytorch 2.1.0, lightning 2.3.0 and previously reported gradient boosting classifiers were taken from: https://github.com/Niopek-Lab/DI_screen. Protein sequence embeddings were computed using ESM-2 (https://github.com/facebookresearch/esm). Multiple sequence alignments were computed with MMseq2 (https://github.com/soedinglab/MMseqs2). Secondary structure prediction was performed using S4pred (https://github.com/psipred/s4pred). Analysis of NGS data was performed using sabre 1.0 and CRISPResso2 2.3.1 (https://github.com/pinellolab/CRISPResso2). The ProDomino model is available on Github: https://github.com/Niopek-Lab/ProDomino.

For manuscripts utilizing custom algorithms or software that are central to the research but not yet described in published literature, software must be made available to editors and reviewers. We strongly encourage code deposition in a community repository (e.g. GitHub). See the Nature Portfolio guidelines for submitting code & software for further information.

Data

Policy information about availability of data

All manuscripts must include a data availability statement. This statement should provide the following information, where applicable:

- Accession codes, unique identifiers, or web links for publicly available datasets
- A description of any restrictions on data availability
- For clinical datasets or third party data, please ensure that the statement adheres to our policy

Additional information, including relevant amino acid sequences, targeted genomic loci and PCR primers for InDel quantification are provided in the supplementary information. Genbank files of DNA constructs are provided as supplementary data file. Important constructs will be shared on Addgene (Add gene ID: 231574 - 231578). Additional data will be shared upon reasonable request. Protein structures for phosphoglycerate kinase (PDB IDs: 4NG4), Rvb1/Rvb2 heterohexamer (RuvB-like 1, PDB IDs: 5OAF), PAC (PDB ID: 7K0A), CAT (PDB ID: 1PD5), SpCas9 (PDB ID: 4UN3) and MbCas12a (PDB ID: 6IV6) were previously reported by others and are available on the RCSB Protein Data Bank (https://www.rcsb.org/).

Human research participants

Policy information about studies involving human research participants and Sex and Gender in Research.

Reporting on sex and gender	N/A
Population characteristics	N/A
Recruitment	N/A
Ethics oversight	N/A

Note that full information on the approval of the study protocol must also be provided in the manuscript.

Field-specific reporting

Please select the one below that is the best fit for your research. If you are not sure, read the appropriate sections before making your selection.

☒ Life sciences ☐ Behavioural & social sciences ☐ Ecological, evolutionary & environmental sciences

For a reference copy of the document with all sections, see nature.com/documents/nr-reporting-summary-flat.pdf

Life sciences study design

All studies must disclose on these points even when the disclosure is negative.

Sample size	No sample size calculation was performed. The sample size was determined based on pilot experiments and aligned to conventions in the field (Benman et al., Nature Methods 22, 2025, https://doi.org/10.1038/s41592-024-02572-4; Ferreira da Silva et al., Nature Biotechnology, 2024, https://doi.org/10.1038/s41587-024-02324-x)
Data exclusions	No experimental data were excluded from the analysis. The protein databases were filtered during pre-processing for our machine learning pipeline, e.g. filtering for sequence identity, as described in the methods section.
Replication	All attempts for replication were successful. The number of replicates performed is indicated in each figure legend, where applicable.
Randomization	No randomization was used, as samples and controls were treated side-by-side using the identical protocols and workflows for analysis. Also, the study did not involve human or animal subjects.
Blinding	No blinding was used, as the majority of data was automatically collected by machines and analyzed by standard workflows.

Reporting for specific materials, systems and methods

We require information from authors about some types of materials, experimental systems and methods used in many studies. Here, indicate whether each material, system or method listed is relevant to your study. If you are not sure if a list item applies to your research, read the appropriate section before selecting a response.

Materials & experimental systems

n/a	Involved in the study	
☒	☐	Antibodies
☐	☒	Eukaryotic cell lines
☒	☐	Palaeontology and archaeology
☒	☐	Animals and other organisms
☒	☐	Clinical data
☒	☐	Dual use research of concern

Methods

n/a	Involved in the study	
☒	☐	ChIP-seq
☒	☐	Flow cytometry
☒	☐	MRI-based neuroimaging

Eukaryotic cell lines

Policy information about cell lines and Sex and Gender in Research

Cell line source(s)	HEK 293T cells were obtained from ATCC (cat. no. CRL-3216)
Authentication	The cell line was authenticated via Single Nucleotide Polymorphism Profiling using a commercially available service (Multiplexion, Heidelberg, Germany) prior to usage.
Mycoplasma contamination	The cell line was tested negative for mycoplasma contamination via a commercially available service (Multiplexion, Heidelberg, Germany) prior to usage.
Commonly misidentified lines (See ICLAC register)	HEK (293T) is a standard cell line widely used for transient transfection and CRISPR/Cas9 experiments.
